# Self-assessment of Articles Published in the Balkan Medical Journal According to Their Study Design with Regards to Impact Factor

**DOI:** 10.4274/balkanmedj.galenos.2020.2020.6.001

**Published:** 2020-10-23

**Authors:** Oğuz KIZILKAYA, Necdet SÜT, Mustafa İNAN

**Affiliations:** 1 Department of Pediatric Surgery, Trakya University School of Medicine, Edirne, Turkey; 2 Biostatistics Editor, Balkan Medical Journal Department of Biostatistics and Informatics, Trakya University School of Medicine, Edirne, Turkey; 3 Deputy Editor, Balkan Medical Journal Department of Pediatric Surgery, Trakya University School of Medicine, Edirne, Turkey

Balkan Medical Journal (Balkan Med J) is a general medical journal that has been published for 41 years. It is managed by the School of Medicine of the Trakya University and has been indexed in the Web of Science (WoS) and PubMed (Index Medicus) since 2007 and 2011, respectively. Despite many difficulties, the impact factor has increased over the years and its popularity is increasing both in the Balkans and worldwide (1,2). To reflect the science produced in the Balkans, a challenging geographical region, the journal management respected ethical values, paid special attention to transparency, and placed emphasis on institutionalism (1). Hence, it has caught the anchor of reputable, internationally recognized initiatives such as ICMJE and EASE. Balkan Med J, which started to publish articles from different parts of the world over time, has attempted to fulfill its increasing responsibilities both regionally and internationally each year.

In this editorial, we aimed to carry out a self-evaluation of Balkan Med J in terms of citations received from the articles published since the indexing in WoS. We examined the citations received and their distribution by study designs. We previously conducted a short analysis of the journal's citations and their sources (3). However, the Journal has never been evaluated bibliometrically to date. We think that this study will shed light on the publication policies of the Journal in the coming period. Moreover, we believe it will show the publication categories to which frequently cited articles belong in the Balkan Med J.

All articles published in the Balkan Med J and the citations received between 2011 and 2019 were scanned in the WoS database. The study designs were written by examining the articles one by one and were noted by OK. The study design of each article was checked according to the research expert NS. Study types containing fewer than 2 articles were combined in the category “Other”. Controlled studies without randomization were classified as non-randomized clinical trials (non-RCT). Cited articles that were withdrawn were excluded from the study. As of July 8, 2020, the top 10 most cited articles in WoS were determined. Kruskal-Wallis test and homogeneous subset methods were used to compare the citations according to the study designs of the articles. A p-value <0.05 was considered statistically significant.

Nine hundred and ten articles published between 2011 and 2019 were reviewed. The three most common types of studies published in the journal were cross-sectional (25.6%), case report (21.9%), and animal experiment (9.1%). The least published study type was meta-analysis/systematic review (0.3%). In the last three years, an increase was observed in the invited review, clinical image, and letter to the editor categories, while the number of printed animal experiments decreased. Also, the number of cross-sectional studies, which is the most popular research type of the journal, decreased in the last 2 years (Table 1). After examining the average number of citations in the WoS directory, it was observed that the top three articles with the highest number of citations were review (5.50), animal experiment (3.71), and randomized controlled trials-RCT (3.05), and the least cited studies were letter to the editor (0.49) and clinical image (0.36). Considering the average number of citations per year, the three most cited articles were review (1.48), animal experiment (0.71), and meta-analysis/systematic review (0.67), and the least cited articles were clinical image (0.13) and letter to the editor (0.11) (Figure 1).

Kruskal-Wallis test revealed a significant difference in the average citations per year among study designs (p<0.001). Homogeneous subsets according to study designs are shown in Table 2. Letter to the editor and clinical image were significantly different from all other study designs except for other, non-RCTs, cohort, and editorial. Furthermore, cross-sectional, case-control, RCTs, animal experiment, meta-analysis/systematic review, and review study designs were significantly different from all of the other study designs except for *in vitro*/cell, diagnostic accuracy, and brief report. The top 10 most cited articles published in Balkan Med J are given in Table 3.

Considering the number of citations received within 2 years after publication showing the contribution of study types to the impact factor, it was found that review studies received the highest number of citations, followed by meta-analysis/systematic review, diagnostic accuracy, and animal experiment studies (Figure 2). The least cited ones were clinical image, cohort, letter to editor, and case report.

Among the articles published in the Balkan Med J in the period 2011-19, the most cited study type was review articles as expected. This is similar to other international journals that analyzed their status (4,5,6,7,8). Invited reviews are also published in the journal. The editor finds articles for this type by inviting researchers with highly cited articles in their field of study to write articles. Carefully selected candidates are asked to share the current status of their work with the reader, and these articles are put into the peer-review process. Our analysis showed that this article type was highly cited, suggesting that the journal management makes accurate decisions in choosing the author and topic title. A similar picture emerged when the top 10 articles in the journal were examined.

Systematic reviews and meta-analyses, accepted as having high scientific value, are also articles with high citation potential (4,5,7). Our study revealed that the Balkan Med J had few printed articles of this type, and those printed were highly cited. While evaluating the results of this study, this important limitation should be taken into account. Nevertheless, the journal management should tell potential authors that they want to publish articles such as meta-analysis and systematic reviews and that they have special expectations for the publication of such articles. It should also enrich the journal’s referee pool with qualified researchers to achieve this goal. Thus, it should guarantee an objective peer-review process with fast evaluation time, free printing, and correct referee assignments for potential authors. For these studies to be carried out effectively, a special group formed by members of the editorial board can focus on meta-analysis and systematic reviews.

Among the individual studies that differ from review and meta-analysis, we found that animal experiment articles had the highest average number of citations per year. We think this may be an interesting point, because at first glance, although clinical studies seem to have higher citation potential, animal experiment articles in Balkan Med J received higher citations. We believe this is because of the increasing importance of pre-clinical studies around the world in recent years given the ethical principles, resulting in a large number of animal experiments. We think that this important finding will ensure that animal experiments submitted to the journal are evaluated from a different perspective and viewed as articles with potential for citation. Also, we believe that successful pre-clinical researchers can be invited to both the author and referee pool by giving special importance to this article type while determining the publication policies and thus, turn the journal into an important attraction. However, our study revealed that the number of animal experiment studies published in the Balkan Med J has started to decrease in recent years. We underline that this situation has the potential to decrease relatively in citation expectation. In the Balkan Med J, we believe that the number of animal experiment studies covering current issues, which are within the framework of ethical rules, well planned, properly written, and evaluated by the expert referees should be increased.

Looking at the homogeneous subsets according to the study designs, it is noteworthy that the annual average citations numbers received by case-control, cross-sectional, and randomized controlled studies published in the Balkan Med J was quite close to each other. The publication of prospective randomized controlled studies will contribute positively to the citation profile. Authors seek to publish such studies in journals of category Q1 and Q2. We believe that Balkan Med J will soon be in the Q2 category, and more prospective randomized controlled studies will be sent to the journal. The search for uncompromising quality publication, which is currently being implemented by the journal management, seems to be the most important guarantor to achieve this goal.

According to the literature, RCT and cohort studies with a high level of evidence are expected to have a greater effect on the impact factor (5,7). However, specific to the Balkan Med J, it was observed that these two types of study designs did not affect the impact factor as expected. We think there may be two reasons for this; either the study topics of such articles published in the Journal were low in popularity, or even though popular studies were published, the message was not found adequately interesting and effective. We believe that the Journal management should pay particular attention to popular study topics and message, specifically when choosing RCT and cohort studies.

We, therefore, think that correctly selected invited reviews and animal experiments have a major role in the current success of Balkan Med J, and the one factor that will determine how the Journal will be positioned in the future will be the ability to publish randomized controlled studies with meta-analyses and systematic reviews. We believe that increasing the recognition of the journal outside the Balkan region and worldwide are the main parameter of sustainable development. For the data obtained from this study to be further developed and remain reliable, we believe that the citations received by the article groups should be compared with those of other journals.

## Figures and Tables

**Table 1:**
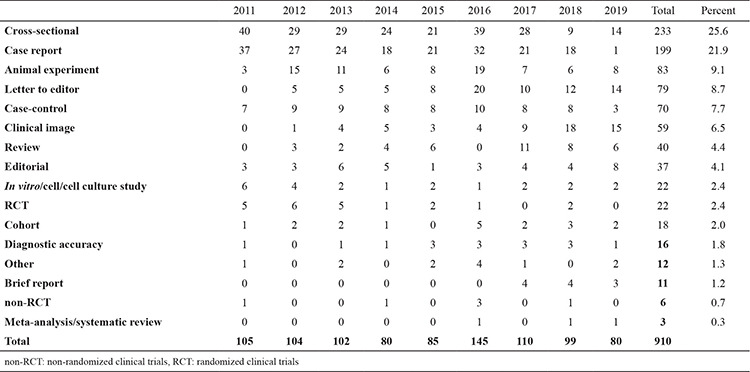
Distribution of publication types by years

**Table 2:**
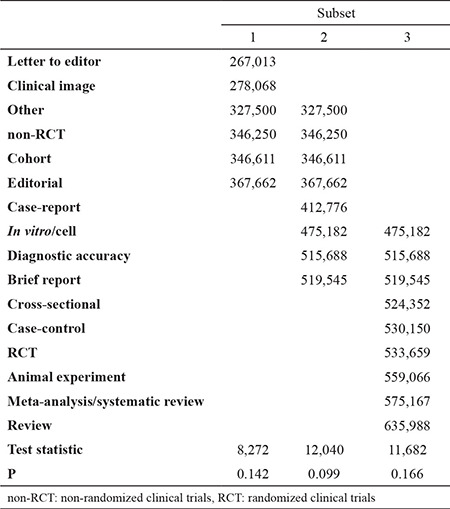
Homogeneous subsets according to the study designs

**Table 3:**
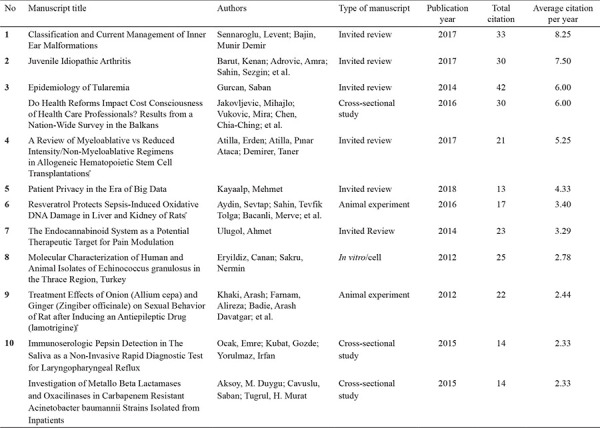
Most cited top 10 articles published in Balkan Medical Journal as of the date of 08.07.2020

**Figure 1:**
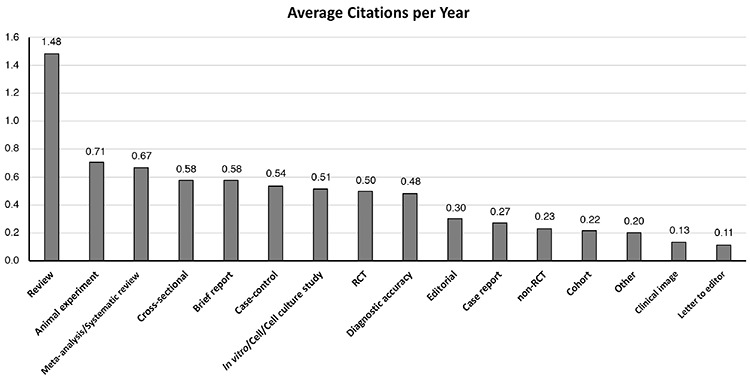
Average citations per year by study designs. RCT: randomized clinical trial

**Figure 2:**
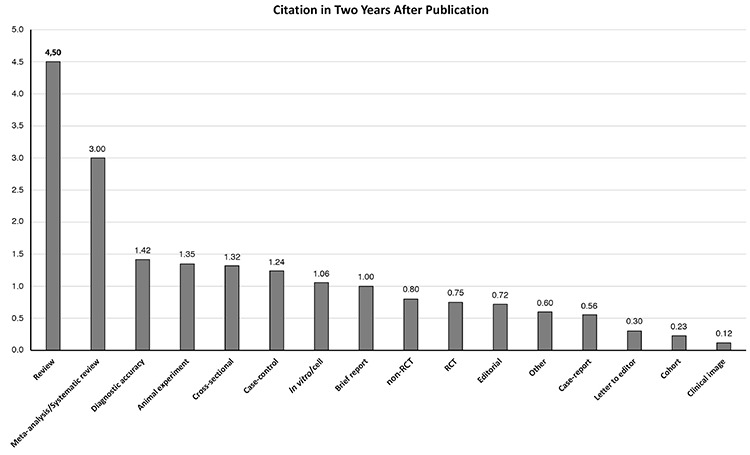
Average citations in two years after publication. RCT: randomized clinical trial

## References

[1] Kocak Z (2020). The impact factor of Balkan Medical Journal continues to rise. Balkan Med J.

[2] İnan M, Erbas H, Kocak Z, Uzun C (2019). Balkan Medical Journal and Legal Regulation. Balkan Med J.

[3] Kocak Z, Sut N, Asan A (2017). Development and progress of Balkan Medical Journal. Balkan Med J.

[4] Kubinger KD, Heuberger N, Poinstingl H (2010). On the self-evaluation of a journal’s impact factor. Psychol Test Assess Model.

[5] Lei Y, Tan BJ, Zou Z, Zhang M, Song R, Qu S, et al (2014). Publication patterns and citation analysis of APJTM during 2008 and 2014. Asian Pac J Trop Med.

[6] Chockattu SJ, Deepak BS (2019). Publication patterns in restorative dentistry and endodontics. Restor Dent Endo.

[7] Allareddy V, Lee MK, Shah A, Elangovan S, Lin Cy (2012). Association between study design and citation counts of articles published in the American Journal of Orthodontics and Dentofacial Orthopedics and Angle Orthodontist. Orthodontics (Chic).

[8] Wolf DM, Williamson PA (2009). Impact factor and study design: The academic value of published research (AVaRes) score. Ann R Coll Surg Engl.

